# Comparison of image quality of two versions of deep-learning image reconstruction algorithm on a rapid kV-switching CT: a phantom study

**DOI:** 10.1186/s41747-022-00314-9

**Published:** 2023-01-09

**Authors:** Djamel Dabli, Maeliss Loisy, Julien Frandon, Fabien de Oliveira, Azhar Mohamad Meerun, Boris Guiu, Jean-Paul Beregi, Joël Greffier

**Affiliations:** 1 Department of Medical Imaging, IMAGINE UR UM 103, Montpellier University, Nîmes University Hospital, Bd Prof Robert Debré, 30029 Nîmes Cedex 9, France; 2 Saint-Eloi University Hospital, Montpellier, France

**Keywords:** Abdomen, Contrast media, Deep learning, Image processing (computer assisted), Phantoms (imaging)

## Abstract

**Background:**

To assess the impact of the new version of a deep learning (DL) spectral reconstruction on image quality of virtual monoenergetic images (VMIs) for contrast-enhanced abdominal computed tomography in the rapid kV-switching platform.

**Methods:**

Two phantoms were scanned with a rapid kV-switching CT using abdomen-pelvic CT examination parameters at dose of 12.6 mGy. Images were reconstructed using two versions of DL spectral reconstruction algorithms (DLSR V1 and V2) for three reconstruction levels. The noise power spectrum (NSP) and task-based transfer function at 50% (TTF_50_) were computed at 40/50/60/70 keV. A detectability index (d') was calculated for enhanced lesions at low iodine concentrations: 2, 1, and 0.5 mg/mL.

**Results:**

The noise magnitude was significantly lower with DLSR V2 compared to DLSR V1 for energy levels between 40 and 60 keV by -36.5% ± 1.4% (mean ± standard deviation) for the standard level. The average NPS frequencies increased significantly with DLSR V2 by 23.7% ± 4.2% for the standard level. The highest difference in TTF_50_ was observed at the mild level with a significant increase of 61.7% ± 11.8% over 40−60 keV energy with DLSR V2. The d' values were significantly higher for DLSR V2 *versus* DLSR V1.

**Conclusions:**

The DLSR V2 improves image quality and detectability of low iodine concentrations in VMIs compared to DLSR V1. This suggests a great potential of DLSR V2 to reduce iodined contrast doses.

**Supplementary Information:**

The online version contains supplementary material available at 10.1186/s41747-022-00314-9.

## Key points

- A deep learning (DL) image reconstruction algorithm is available for rapid kV-switching computed tomography.
- This new DL spectral reconstruction increases lesion detectability on virtual monoenergetic images.
- This new DL spectral reconstruction may reduce the dose of iodinated contrast medium administered in patients.

## Background

The proportion of computed tomography (CT) examinations using contrast media is about 40% worldwide [1]. However, contrast medium administration may induce adverse events in some patients, such as acute kidney injury, particularly in patients with impaired renal function [2, 3]. Optimising the amount of iodined contrast media injected is therefore a challenge [4, 5].

The latest advances in CT technology offer great potential to improve image quality and thus reduce the radiation dose and the amount of contrast medium administered [6, 7]. Among these developments, dual-energy CT (DECT) is of interest to optimise the amount of iodine injected [8–10]. Indeed, this technique offers a capacity to enhance the contrast of lesions using specific images such as virtual monoenergetic images (VMIs) [11–14] as compared to conventional CT images with single-energy (SECT). In addition, the use of iodine-specific images allows the radiologist to quantify the iodine concentration, which improves the characterisation of lesions in abdominal imaging [15–19]. To obtain this type of images, DECT is based on the acquisition of two x-ray spectra, low and high-energy. A material decomposition algorithm is then used to characterise different materials based on low- and high-density base materials (*e.g.,* water and iodine) [20]. Several DECT platforms have been developed with different acquisition and detection methods [21] and thus different spectral performances [22–25]. One of them, developed by Canon Medical Systems Corporation is based on the rapid kV-switching technique. The system switches from high (135 kVp) to low (80 kVp) with duration lower than 1 ms during acquisition. The material decomposition occurs in the raw data and in the image domains [26].

In addition, deep learning (DL) image reconstruction algorithms were recently developed for conventional SECT [27–30] and more recently also for DECT platforms [26, 31, 32]. For the Canon Medical DECT platforms, a DL-based spectral reconstruction (DLSR) is available, based on the creation of deep learning views generated by the trained neural network for opposite and for the same energy views [31, 33]. These energy views are generated by transforming the views from one energy to the other [26, 31]. The measured views are completed by DL views at each energy to create a full sinogram for each kV. The spectral reconstruction was trained on complete sinograms obtained at each energy using different patient and phantom data (more details are provided in Supplementary material). This DLSR was shown to have the capacity to generate low-noise spectral images [26, 31].

The first version of DLSR developed by Canon Medical Systems Corporation in 2019 allowed one slice thickness reconstruction of 0.5 mm and a body spectral kernel [31]. A new release of this algorithm was introduced in 2021, also allowing reconstruction with slice thickness of 0.5 mm. Between the first version of DLSR and the second, the neural network was trained with a large number of new datasets from patients and phantoms. This training with high quality and high quantity datasets led to the improvement of the algorithm performance and thus of the quality of the spectral images obtained. Several studies have assessed the performance of the first version of this DLSR [26, 31] and one study has compared its latest version with four other DECT platforms equipped with iterative reconstruction algorithms [31]. However, to our knowledge, no study has yet compared the performance of the two versions of this DLSR and their potential for reducing the iodined contrast media doses. This comparison is important from a clinical point of view in order to assess the potential gain in image quality and improvement of lesion detection that can be expected from this version for contrast-enhanced abdominal CT examinations. If an improvement of the image quality is observed on the phantom, a validation on patient will be necessary in a second step.

Thus, the purpose of our study was to assess the impact of the new version of the DLSR on image quality at low energy levels of VMIs. A task-based image quality assessment (noise magnitude, noise texture, spatial resolution, and detectability of low iodine concentration) was performed.

## Methods

### Phantoms

**Fig. 1:**
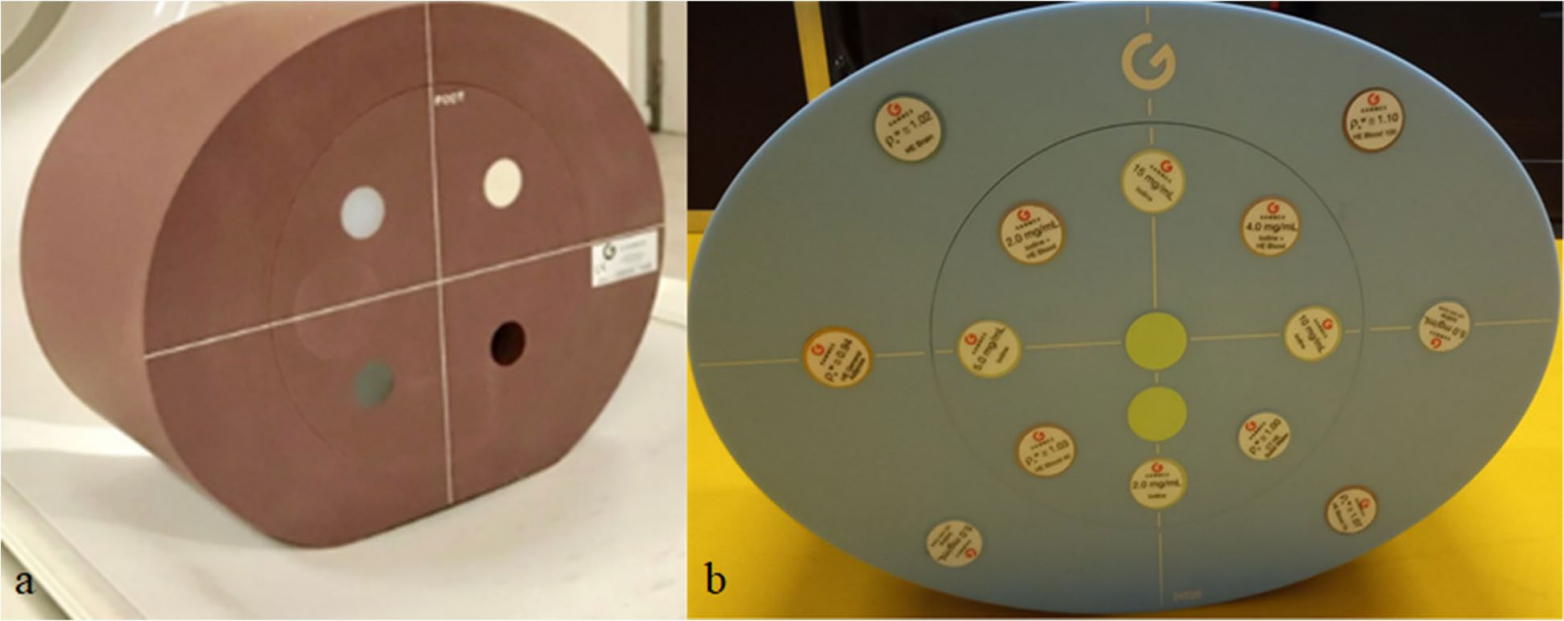
Images of the phantoms used: **a** ACR CT 464 phantom. **b** Multienergy CT phantom. *CT* Computed tomography

The 20-cm diameter American College of Radiology (ACR) Quality Assurance phantom (Gammex 464, Gammex, Middleton, USA) was scanned to assess the image quality by measuring the noise power spectrum (NPS) and task-based transfer function (TTF). This phantom was placed in its elliptical ring (26 × 33 × 16 cm^3^) to simulate the abdomen (Fig. 1a). A multienergy CT phantom (Multi-Energy CT phantom, Sun Nuclear, Middleton, USA) associated with its elliptical body insert (30 × 40 × 15 cm^3^) was also used to assess the contrast between iodine and solid water inserts (Fig. 1b).

### CT scanners and scanning protocols

All acquisitions were performed on the Aquilion ONE PRISM Edition CT system (Canon Medical Systems, Otawara, Japan). Both phantoms were scanned three times with the DECT mode using the same clinical protocol for abdomino-pelvic examinations: a 80/135 kVp switching, a collimation of 80 × 0.5 mm, a rotation time of 1 s and a pitch value of 0.813. The tube current was set at 230 mA to obtain a CT volume dose index of 12.6 mGy, close to the French national diagnostic reference level for abdomen and pelvis examinations fixed at 13 mGy in France [34].

Raw data were reconstructed using the two versions of the DLSR (DLSR V1 and DLSR V2), the “Body spectral” reconstruction kernel, and using the three available DLSR levels: mild, standard, and strong. As the DLSR did not allow a 1-mm thick reconstruction, all raw data were reconstructed using the 0.5-mm slice thickness and a 0.5-mm increment for both DLSR versions. The field of view used was 250 mm for the ACR phantom, and 420 mm for the multienergy CT phantom.

For each acquisition, VMIs were reconstructed using the Vitrea workstation (Canon Medical Informatics, Minnetonka, Minnesota, USA) at four low energy levels (40, 50, 60, and 70 keV) used in clinical practice to improve the iodine contrast.

### Assessment of iodine contrast on VMIs

**Fig. 2:**
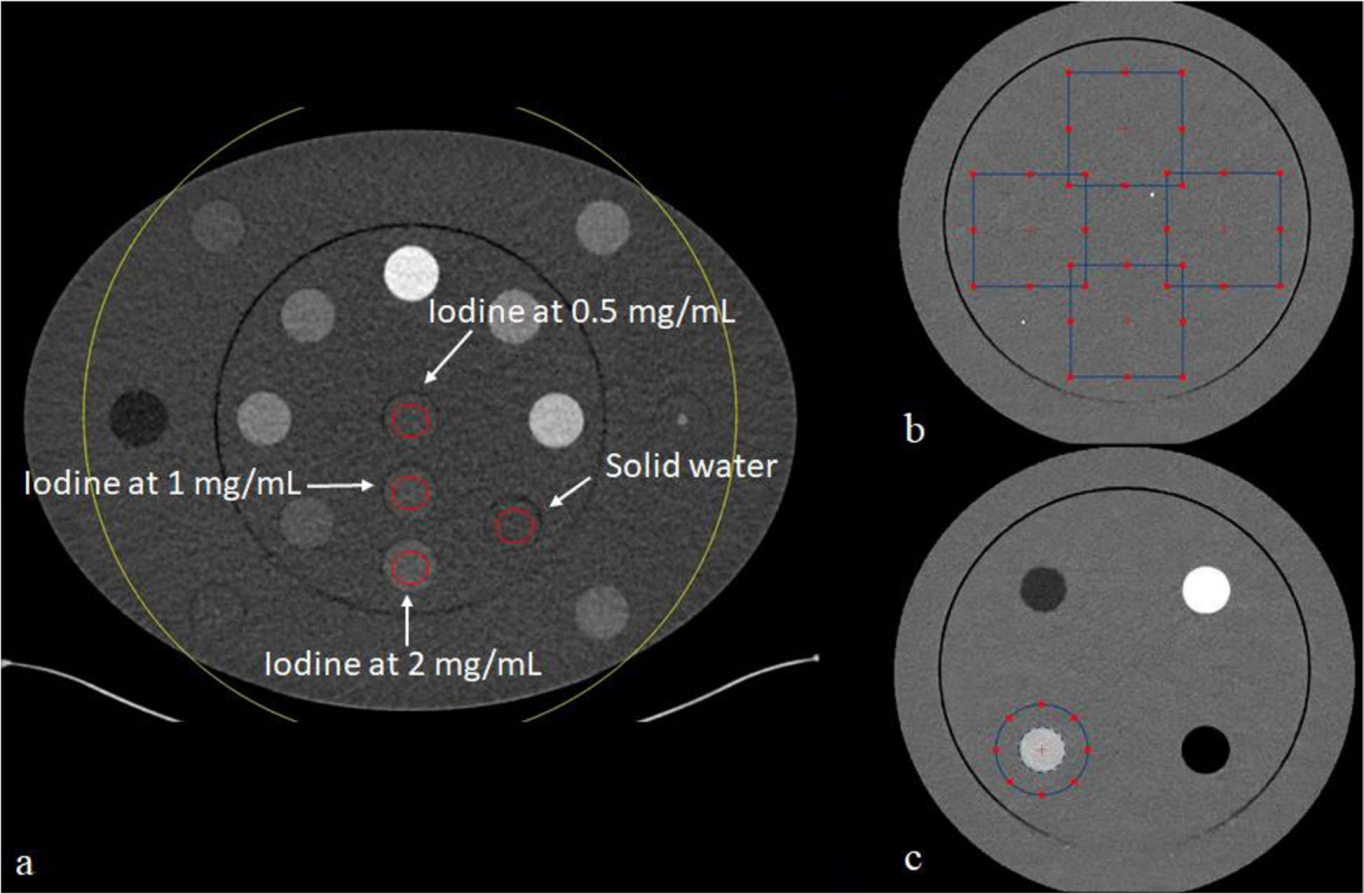
Regions of interest (ROIs) placement on the images. **a** ROI to measure the iodine contrast relative to solid water in the multienergy phantom. **b** ROI placed on the uniform module of the ACR phantom to compute the noise power spectrum (NPS). **c.** ROI placed on the acrylic insert of the ACR phantom to compute the task-based transfer function

For each DLSR level and for both DLSR versions, the HU values were measured in the central slice of the multienergy CT phantom. One circular region of interest (ROI) of 2-cm diameter was placed on the solid water insert and on three iodine inserts with an iodine concentration of 2, 1 and 0.5 mg/mL (Fig. 2a). For each insert, the mean HU value within each ROI was computed for the VMIs at 40, 50, 60, and 70 keV.

The contrast between the iodine and solid water inserts was calculated at each energy level for all DLSR levels and for both DLSR versions according to the following formula:

1 $$Contrast={HU}_{iodine}-{HU}_{solid\ water}\kern0.5em$$

where, *HU*_*iodine*_corresponds to the mean HU value of each iodine insert (0.5, 1.0, and 2.0 mg/mL) and *HU*_*solid water*_ to the solid water value.

### Task-based image quality assessment on VMIs

A task-based image quality assessment was performed using the ImQuest software (version 7.1, Duke University, USA) to assess the noise magnitude and texture using the NPS and the spatial resolution using the TTF [35, 36]. The detectability index (d′) was computed to assess the ability of the radiologist to detect enhanced lesions. All these metrics were calculated for three DLSR levels for the two versions, and for all VMI levels (40, 50, 60, and 70 keV).

#### Noise power spectrum

For each DLSR level and for both spectral versions, the NPS was computed on the uniform module of the ACR phantom using 40 consecutive slices. Four square ROIs of 128 x 128 pixels were placed in this uniform module (Fig. 2b) and the NPS was calculated following this formula:

2 $${NPS}_{2D}\left({f}_x,{f}_y\right)=\frac{\Delta _x{\Delta }_y}{L_x{L}_y}\frac{1}{N_{ROI}}\sum_{i=1}^{N_{ROI}}{\left|{FFT}_{2D}\left\{{ROI}_i\left(x,y\right)-{FIT}_i\left(x,y\right)\right\}\right|}^2$$

where *Δ*_*x*_ and *Δ*_*y*_ are the pixel sizes in the x- and y-directions; *FFT* is the Fast Fourier Transform; *L*_*x*_ and *L*_*y*_ are the lengths of the ROIs in the x- and y-directions; *N*_*ROI*_ is the number of ROIs; *ROI*_*i*_(*x*, *y*) is the mean pixel value measured for a ROI at the position (x, y) and *FIT*_*i*_(*x*, *y*) is a second order polynomial fit of *ROI*_*i*_(*x*, *y*). The noise magnitude and the average spatial frequency (f_av_) were calculated to quantify the noise level and noise texture respectively. A f_av_ at low spatial frequencies may indicate a blotchy noise appearance. The following formula was used to compute the f_av_ values:

3 $${f}_{av}=\frac{\int f. NPS(f) df}{\int NPS(f) df}$$

where *f* is the radial spatial frequency and *NPS*(*f*) is the radially re-binned/average 1D NPS [36].

#### Task-based transfer function

The TTF was assessed using the acrylic insert of the ACR phantom (Fig. 2c) following the methodology reported by Richard et al. [37]. A circular ROI was placed around the insert and a circular-edge technique was used to measure the edge spread function, which was obtained by calculating the radius of each pixel from the centre of each pixel of the insert. The line spread function was obtained by derivation of the edge spread function. The TTF was then computed from the line spread function normalised Fourier transformation. It was computed using 20 consecutive slices.

#### Detectability index

Three task functions were defined to model the detection of enhanced lesions of 10 mm diameter with low iodine concentrations of 0.5, 1, and 2 mg/mL. The TTF results for the acrylic insert were used for each detection task combined with the NPS to calculate the detectability index (d′) using a non-prewhitening observer model with an eye filter [38]:

4 $${d^{\prime}}_{NPWE}^2=\frac{{\left[\iint \left|W\left(u,v\right)\right|2. TTF{\left(u,v\right)}^2.E{\left(u,v\right)}^2 dudv\right]}^{{}^2}}{\iint {\left|W\left(u,v\right)\right|}^2. TTF{\left(u,v\right)}^2. NPS{\left(u,v\right)}^2.E{\left(u,v\right)}^4 dudv}$$

where *u* and *v* are the spatial frequencies in the x- and y-directions, *E* the eye filter that models the human visual system sensitivity to different spatial frequencies, and *W(u,v)* the task function defined as:

5 $$W=\left|F\left\{{h}_1\left(x,y\right)-{h}_2\left(x,y\right)\right\}\right|$$

where *h*_1_(*x*, *y*) and *h*_2_(*x*, *y*) correspond to the object present and the object absent hypotheses.

The contrast of each clinical task was measured directly on the iodine inserts of the multienergy CT phantom for each corresponding iodine concentration and for each energy level. The reading conditions used to obtain d’ were a 1.5 zoom factor, a viewing distance of 500 mm, a 300-mm field of view and a 0.05-mm pixel size.

### Statistical analysis

Data are given as means and standard deviations. All quantitative data were compared between DLSR V1 and DLSR V2 using the Wilcoxon test for appeared samples. A *p*-value lower than 0.05 was considered significant.

## Results

### Assessment of iodine contrast values on VMIs

For the DLSR V1, the measured contrast between iodine and solid water inserts (Table 1) decreased when the energy level increased for all DLSR levels and all iodine concentrations. The greatest difference in contrast values as function of the DLSR levels were observed with 0.5 mg/mL iodine at 50 and 60 keV, and a non-significant difference of 16.0% ± 6.1% (mean ± standard deviation) (*p* = 0.224) was observed between the standard and mild levels for 50 keV.

**Table 1:** Contrast values of the iodine insert obtained for the low energy levels on virtual monoenergetic images (VMIs) and with the three reconstruction levels of both versions of the deep learning spectral reconstruction (DLSR V1 and DLSR V2)

Iodine concentration	Energy level	DLSR V1			DLSR V2		
		Mild	Standard	Strong	Mild	Standard	Strong
2 mg/mL	40 keV	138 ± 4.0	142 ± 2.5	143 ± 3.0	148 ± 5.1	149 ± 3.6	155 ± 5.0
	50 keV	97 ± 1.9	101 ± 2.0	96 ± 2.8	98 ± 2.2	102 ± 4.0	98 ± 3.5
	60 keV	69 ± 1.7	67 ± 1.0	67 ± 1.5	70 ± 1.9	68 ± 2.8	70 ± 3.1
	70 keV	46 ± 1.2	45 ± 0.8	44 ± 1.0.	49 ± 1.1	48 ± 1.0	48 ± 1.4
1 mg/mL	40 keV	83 ± 5.0	89 ± 8.0	86 ± 4.5	80 ± 4.5	81 ± 6.4	80 ± 5.1
	50 keV	54 ± 2.8	53 ± 7.0	51 ± 4.1	52 ± 3.2	49 ± 3.3	48 ± 3.8
	60 keV	35 ± 1.6	38 ± 5.0	35 ± 2.8	34 ± 2.0	35 ± 2.4	33 ± 2.9
	70 keV	22 ± 1.0	24 ± 3.7	23 ± 1.0	23 ± 1.8	22 ± 1.2	26 ± 1.5
0.5 mg/mL	40 keV	42 ± 4.6	43 ± 4.7	41 ± 1.8	40 ± 6.0	39 ± 4.3	37 ± 3.3
	50 keV	25 ± 1.5	29 ± 3.4	24 ± 1.5	22 ± 2.0	26 ± 2.2	21 ± 2.1
	60 keV	20 ± 1.0	23 ± 1.8	21 ± 1.2	18 ± 1.4	20 ± 1.6	19 ± 1.8
	70 keV	15 ± 1.3	14 ± 1.1	17 ± 0.8	13 ± 1.8	12 ± 1.2	15 ± 1.0

Data are given as means ± standard deviations

For the DLSR V2, the contrast values decreased when the energy levels increased. The highest difference as function of the DLSR level but not statistically significant was observed with 0.5 mg/mL iodine at 50 keV between the standard and mild levels (18.2% ± 2.4%).

The differences between the contrast values of DLSR V1 and DLSR V2 for iodine concentrations of 1 and 2 mg/mL were not significant (lower by 10%) for all energy and DLSR levels (*p* = 0.653). The greatest differences were obtained for 0.5 mg/mL with significant differences of more than 10% for all energy and DLSR levels (*p* ≤ 0.012). A tendency to lower contrast values with the DLSR V2 was observed at 1 and 0.5 mg/mL iodine concentrations, and the opposite pattern at 2 mg/mL.

### Noise power spectrum

#### Noise magnitude

The noise magnitude (Table 2) decreased significantly from 40 to 70 keV by a mean of 87.8% ± 0.4% for DLSR V1 and 80.0% ± 1.0% in DLSR V2, for all reconstruction levels. For DLSR V1, the noise magnitude values decreased significantly when the DLSR levels increased from mild to strong level. The noise magnitude decreased similarly for all energy levels, by -14.3% ± 1.0% between the mild and standard levels, by -36.5% ± 2.0% between the mild and strong levels. A similar pattern was observed with DLSR V2, with mean decreases of -18.1% ± 1.5% and -36.0% ± 2.4% respectively. For all DLSR levels, the noise magnitude was significantly (*p* ≤ 0.004) lower with DLSR V2 compared to DLSR V1 between 40 and 60 keV. The decrease was similar for these energy levels by a mean of -32.6% ± 1.7%, -36.5% ± 1.4% and 31.8% ± 2.1% for the mild, standard and strong levels, respectively. The noise magnitude values were similar for the two DLSR versions with a no significant difference from 0.0% to 5.2% at 70 keV and *p*-values from 0.250 to 1.000.

**Table 2:** Noise magnitude obtained for the low energy levels on virtual monoenergetic images (VMIs) and three reconstruction levels of both versions of deep learning spectral reconstruction (DLSR)

DLSR version	Energy level	Reconstruction level		
		Mild	Standard	Strong
DLSR V1	40 keV	100.2 ± 0.40	86.6 ± 0.35	65.6 ± 0.31
	50 keV	52.4 ± 0.32	45.3 ± 0.22	34.3 ± 0.28
	60 keV	24.3 ± 0.09	21.0 ± 0.13	15.5 ± 0.19
	70 keV	12.5 ± 0.10	10.6 ± 0.08	7.7 ± 0.12
DLSR V2	40 keV	68.5 ± 0.20	55.1 ± 0.27	44.6 ± 0.41
	50 keV	34.3 ± 0.10	28.1 ± 0.14	22.7 ± 0.21
	60 keV	16.6 ± 0.04	13.6 ± 0.07	10.9 ± 0.10
	70 keV	13.1 ± 0.04	10.6 ± 0.05	8.1 ± 0.08

Data are given as mean ± standard deviation

#### Noise texture

**Fig. 3:**
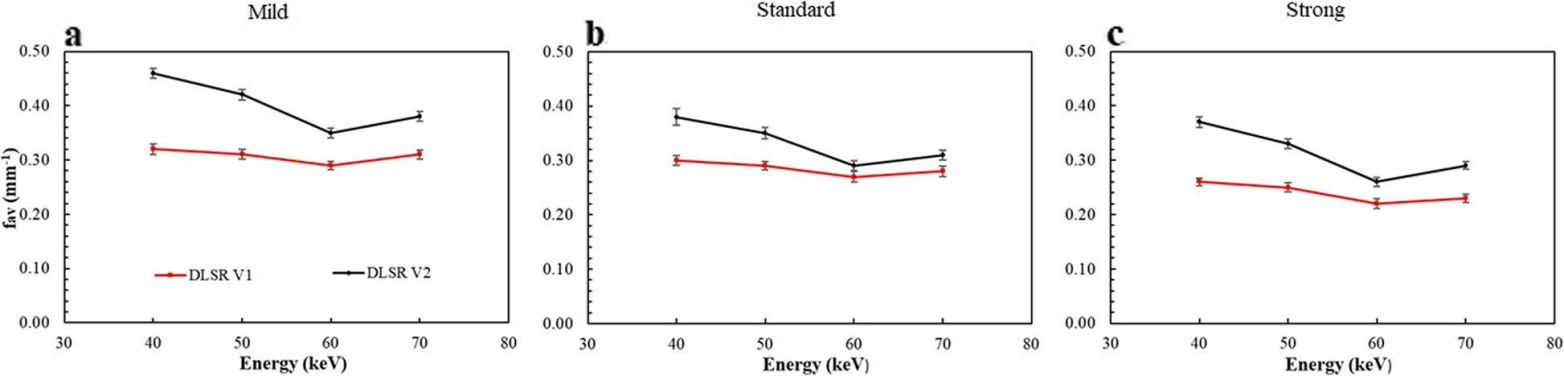
Average noise spectrum frequencies (f_av_) as for three levels of the deep learning spectral reconstruction (DLSR) versions (DLSR V1 and DLSR V2). **a** Mild level. **b** Standard level. **c** Strong level

The average NPS spatial frequency (f_av_) as function of energy levels for both DLSR versions and all DLSR levels are depicted in Fig. 3. For DLSR V1, the f_av_ values were similar between 40 and 50 keV for all DLSR levels (mean difference of -3.4% ± 0.4%; *p* = 0.501). It tended to decrease from 50 to 60 keV by -8.4% ± 3.1% for all DLSR levels. This decrease was not statistically significant (*p* = 0.250). The f_av_ values were similar between 60 and 70 keV for the mild and standard levels (mean difference of 4.12% ± 0.6%; *p* = 0.250) and tended to increase for strong level (6.4% ± 0.8%) but not significantly (*p* = 0.205). For DLSR V2, the f_av_ values decreased significantly from 40 to 60 keV for all DLSR levels in similar proportion (mean decrease of -26.7% ± 3.4%; *p* = 0.004). Between 60 and 70 keV, f_av_ values were similar for mild and standard levels (3.4% ± 0.3%; *p* = 0.270) and increased slightly for strong level (6.0% ± 0.6%) without statistical significance (*p* = 0.250). The f_av_ values were significantly higher with DLSR V2 than with DLSR V1 (*p* ≤ 0.035), particularly at low energy levels. For 40 and 50 keV, the f_av_ values increased by 39.6% ± 5.8% (mild level), 23.7% ± 4.2% (standard level), and 37.2% ± 7.2% (strong level) with DLSR V2 compared with DLSR V1.

### Task-based transfer function

**Fig. 4:**
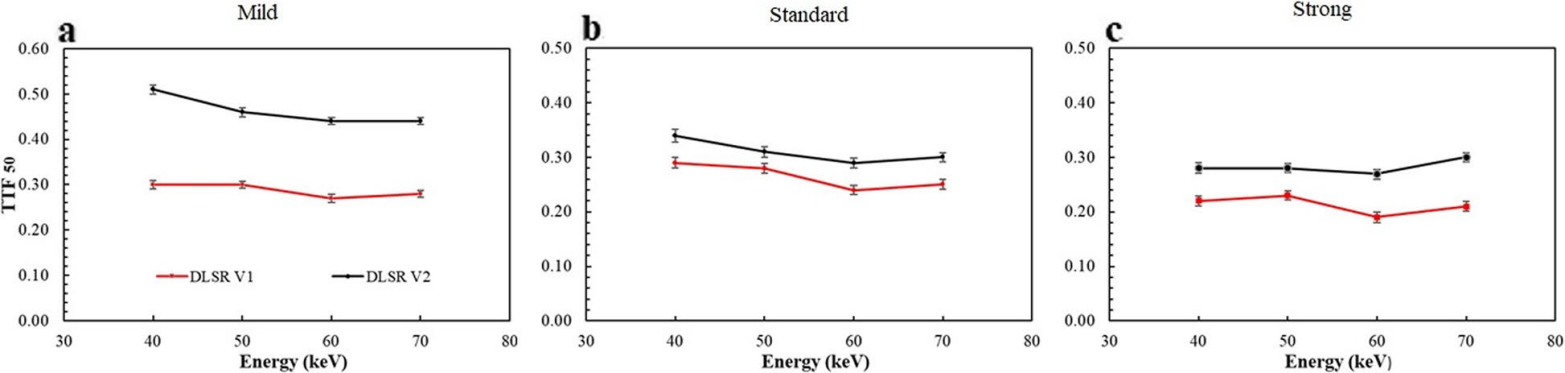
Task-based transfer function at 50% (TTF_50_) as function of the energy levels for three levels of deep learning spectral reconstruction (DLSR) versions (DLSR V1 and DLSR V2). **a** Mild level. **b** Standard level. **c** Strong level

The TTF values at 50% (TTF_50_) as function of the energy level and of the reconstruction level, for both DLSR versions are depicted in Fig. 4. For DLSR V1, the TTF_50_ values tended to decrease from 0.30 ± 0.01 mm^-1^ to 0.28 ± 0.01 mm^-1^ (*p* = 0.250) between 40 and 70 keV at the mild level and from 0.29 ± 0.01 mm^-1^ to 0.25 ± 0.01 mm^-1^ (*p* = 0.250) at the standard level. At the strong level, the TTF_50_ tended to increase between 40 and 50 keV, then decreased from 0.23 ± 0.01 mm^-1^ to 0.21 ± 0.01 mm^-1^ between 50 and 70 keV (p = 0.250). All of these differences were not statistically significant (*p* ≥ 0.250).

For DLSR V2, the TTF_50_ values also tended to decrease from 0.51 ± 0.01 to 0.44 ± 0.01 mm^-1^ (*p* = 0.157) with the mild level and from 0.35 ± 0.01 mm^-1^ to 0.30 ± 0.01 mm^-1^ with the standard level (*p* = 0.281). At the strong level, the TTF_50_ values were similar between 40 and 60 keV (*p* = 0.222) and a tendency to increase was observed from 0.27 ± 0.01 mm^-1^ to 0.30 ± 0.01 mm^-1^ (*p* = 0.250) between 60 and 70 keV. All of these differences were not statistically significant (*p* = 0.157).

For all DLSR and energy levels, the TTF_50_ values increased between DLSR V1 and DLSR V2, with different rates. The greatest difference was observed at the mild level, with a mean significant increase of 61.7% ± 11.8% over energy from 40 to 60 keV (*p* = 0.004) and of 60.1% ± 4.1% over energy from 60 to 70 keV (*p* = 0.031). At these same keV, a significant increase of 24.5% ± 3.9% (*p* = 0.009) and 42.5% ± 0.5% (*p* = 0.031), respectively, was observed at the strong level and of 10.5% ± 0.3% (*p* = 0.004) and of 24.4% ± 5.1% (*p* = 0.031) respectively, for the standard level.

### Detectability indexes

**Fig. 5:**
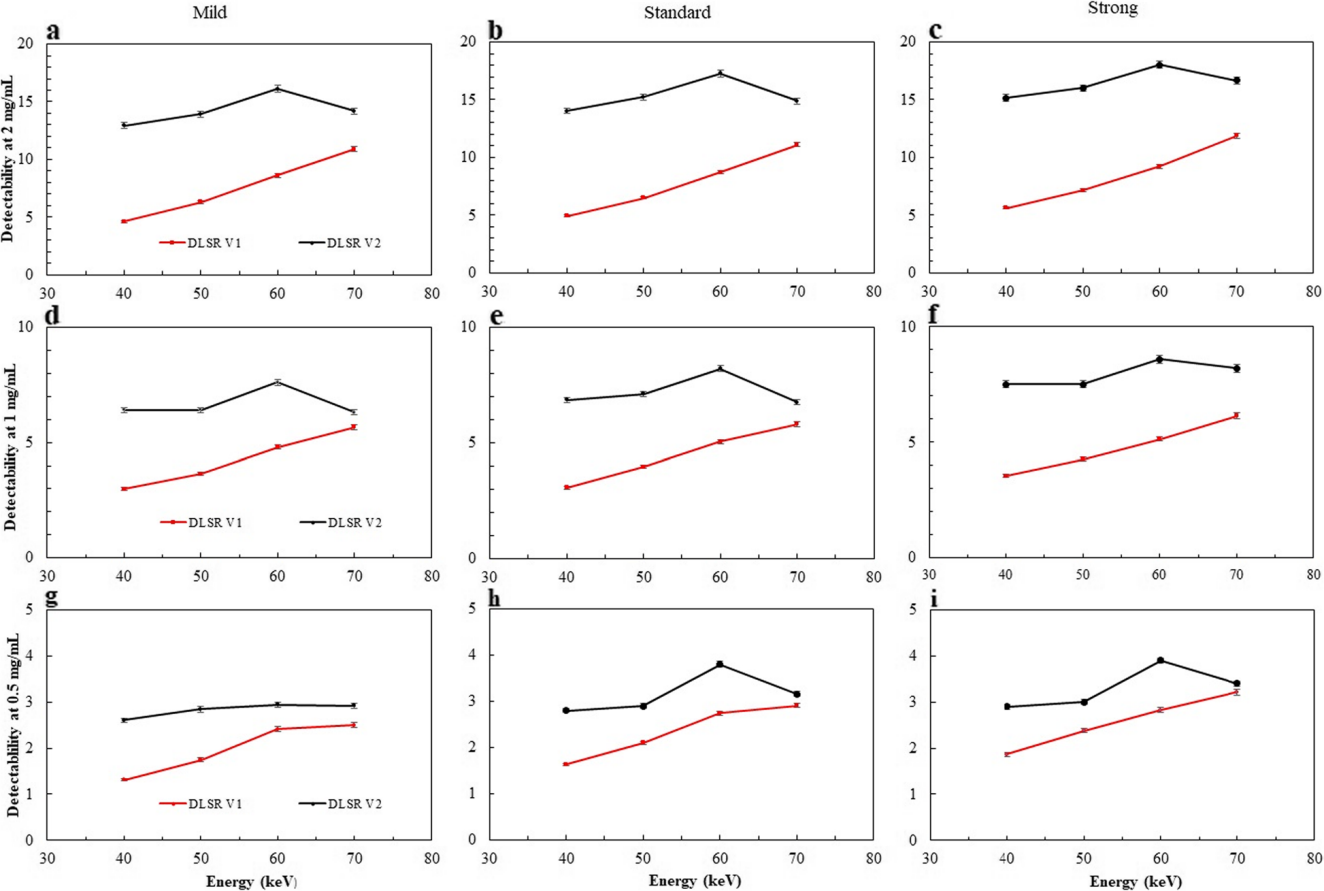
Detectability index as function of the energy levels for three levels of deep learning spectral reconstruction (DLSR) versions (DLSR V1 and DLSR V2) and three iodine concentrations. **a** Mild level, 2 mg/mL iodine. **b** Standard level, 2 mg/mL iodine. **c** Strong level, 2 mg/mL iodine. **d** Mild level, 1 mg/mL iodine. **e** Standard level, 1 mg/mL iodine. **f** Strong level, 1 mg/mL iodine. **g** Mild level, 0.5 mg/mL iodine. **h** Standard level, 0.5 mg/mL iodine. **i** Strong level, 0.5 mg/mL iodine

The d’ values obtained for the three simulated contrast-enhanced lesions are depicted on Fig. 5 as function of the energy level, DLSR level and DLSR version.

For DLSR V1, the highest d’ value was obtained at 70 keV for all DLSR levels and all iodine concentrations. For all DLSR levels, d’ increased significantly between 40 and 70 keV by 80.6% ± 9.3% (*p* ≤ 0.001) at the 0.5-mg/mL iodine concentration, by 84.1% ± 9.7% (*p* ≤ 0.001) at 1 mg/mL and by 124.4% ± 12.3% at 2 mg/mL (*p* ≤ 0.001). The d’ values increased significantly (*p* ≤ 0.001) as the DLSR levels increased for all energy levels, particularly at 0.5 mg/mL with the strong level where d’ increased by 30.8% ± 10.7% compared to the mild level.

For DLSR V2, d’ peaked at 60 keV for all DLSR levels and iodine concentrations. It increased significantly (*p* ≤ 0.001) between 40 and 60 keV by a mean of 27.8% ± 12.7%, 17.7% ± 2.8%, 22.2% ± 2.9% for the 0.5, 1 and 2 mg/mL iodine concentrations, respectively. The d’ values increased significantly (*p* ≤ 0.001) also along with the DLSR level; a mean increase of 16.6% ± 11.6% was observed at 0.5 mg/mL iodine concentration with the strong level compared to the mild level.

The d’ values obtained with DLSR V2 were significantly higher (*p* ≤ 0.001) than with DLSR V1 for all reconstruction and energy levels. This increase was higher with the strong level and decreased when the energy level increased and when the iodine concentration decreased.

## Discussion

To the best of our knowledge, this study is the first to compare the performance of two versions of a DLSR algorithm used in a rapid kV-switching dual-energy platform developed by Canon Medical Systems. The noise magnitude and characteristics, spatial resolution, and detectability in VMIs of three contrast-enhanced lesions at low iodine concentrations were compared. One of the main findings was that the last version of DLSR (V2) presented a lower noise magnitude for energy levels above 70 keV, with an improved noise texture and spatial resolution for all energy and reconstruction levels. In addition, the detectability index was significantly higher with DLSR V2 for all iodine concentrations tested (2.0, 1.0 and 0.5 mg/mL).

The iodine contrast outcomes showed that the difference between iodine contrasts obtained with DLSR V1 and DLSR 2 was significantly higher at 0.5 mg/mL than at 1 and 2 mg/mL iodine. Indeed, the contrast values with the 1 and 2 mg/mL iodine inserts were similar between the two versions. The contrast values of the 0.5 iodine inserts measured with DLSR V1 was significantly lower than with DLSR V2. These results suggest that DLSR versions have an impact on contrast values at very low iodine concentrations. However, the impact of this contrast on the detectability at low iodine concentrations is limited because it also depends on the noise and spatial resolution.

The noise magnitude increased when the energy level decreased with both DLSR versions, in line with the results of different DECT platforms reported in previous studies [9, 23, 31]. It may be explained by the higher attenuation reported at low energy levels. The higher contribution of the photoelectric effect at low energy levels decreases the signal-to-noise ratio on the basis material images. This is related to the introduction of anti-correlated noise [39] during material decomposition used to generate VMIs. In both versions, the noise magnitude decreased as the DLSR level increased. However, the noise magnitude was significantly lower with DLSR V2 compared to DLSR V1 for energy levels between 40 and 60 keV, and it was similar at 70 keV. This result is of major clinical interest because the recommended energy levels for abdominal imaging are specifically those between 40 and 60 keV [40]. However, the use of these low energy levels is often limited by the higher noise in keeping with our findings on reported noise magnitude. The improved noise magnitude obtained with DLSR V2 at low energy levels increases the potential for use of these recommended levels.

Our results also showed that the f_av_ values shifted towards lower frequencies as the DLSR level increased for both versions and shifted towards higher frequencies when using DLSR V2 compared to DLSR V1 for a given reconstruction level. Greffier et al [41] obtained similar results with a SECT between the two versions of the Advance Intelligent Clear-IQ Engine deep learning image reconstruction algorithm (V8 and V10). As for the SECT algorithm, the noise texture in our study changed according to the DLSR level used in both versions; indeed, the DLSR V2 version showed a reduced smoothing effect on the images than DLSR V1. The modification of the image texture between DLSR V1 and DLSR V2 was significant for all energy and reconstruction levels particularly at the mild and strong levels compared to the standard level. The DLSR V2 thus allows reducing noise while improving noise texture for energy levels between 40 and 60 keV. This increases the possibility of using these energy levels without adversely affecting the diagnostic quality.

Our results showed that the TTF_50_ values tended to decrease for both versions when the energy level increased from 40 to 60 keV but not significantly and were similar between 60 and 70 keV for the mild and standard levels. Similar results were obtained by Greffier et al [31] at the standard level with DLSR V2 and a dose level of 10 mGy. A different pattern was observed with the strong level, where the TTF_50_ obtained with DLSR V1 tended to increase from 40 to 50 keV, then to decrease from 50 to 60 keV and to increase beyond. With DLSR V2 at the strong level, the TTF_50_ values were similar between 40 and 60 keV and increased significantly at 70 keV. These results could be explained by the strong noise reduction at the strong level affecting the low frequencies of the signal representing the details of the image that may be considered as noise and thus may be removed. In all cases, the TTF_50_ values obtained with DLSR V2 were significantly higher than with DLSR V1 and for all reconstruction levels, implying an improvement of the spatial resolution in clinical practice with the new DLSR version, particularly for the mild level.

The improvement of noise magnitude, noise texture, and spatial resolution with DLSR V2 led to a higher detectability index for the three simulated enhanced lesions despite a slight decrease in contrast with DLSR V2 at 0.5 mg/mL iodine concentration. However, improvement of the detectability index is dependent on the energy levels and the iodine concentrations. The greatest improvement for V2 was obtained for energy levels of 40 to 60 keV whereas the lower improvement was noted at 70 keV for the three iodine concentrations. Then, the improvement in detectability at 70 keV was probably related to the higher TTF_50_ and f_av_ values obtained with DLSR V2. Our results also showed that the detectability indexes for DLSR V1 peaked at 70 keV, equivalent to a SECT at 120 kVp, for all iodine concentrations at 60 keV with DLSR V2.

These findings imply that the new V2 version of DLSR can improve the detectability of low-enhanced lesions using a lower energy level with a standard radiation dose level equivalent to the national diagnostic reference level. This result is of major interest to reduce the amount of iodine used in patients with acute kidney injuries [3, 42, 43].

Our results showed a strong potential of the new version of DLSR to reduce the amount of iodine injected to the patients when using the 60 keV energy level. Indeed, several studies reported low iodine concentrations (lower than 5 mg/mL) measured for abdominal lesions [13–19]. The detection of these lesions is challenging, requiring higher iodine concentrations and/or radiation doses. This potential can be improved using the DLSR V2 at the strong level. Indeed, this reconstruction level produced less noise than the mild and standard levels but its use in clinical practice with DLSR V1 is limited by the radiological image perception and smoothing as reported for the SECT in a previous study [44].

This improvement in iodine detectability on VMIs implies an improvement in the visualisation and delineation of iodine-enhanced lesions in various abdominal clinical applications [40]. For liver lesions, it improves the detection of hypovascular liver lesions and the diagnostic confidence for hepatic metastases [40]. It may also improve the depiction of intrahepatic veins at low contrast conditions [45]. For pancreas applications, the increase of vascular and parenchymal enhancement using DLSR V2 could increase the detection of small lesions (< 2 cm) which are challenging due to suboptimal contrast conditions [46]. For kidney applications, Patel et al [47] reported a better renal lesion demarcation using VMIs at low keV. The DLSR V2 could improve this demarcation even at low iodine contrast conditions. The DLSR V2 could bring an added value also for studying the bowel by improving the focal hypoenhancement using VMIs at low keV for early detection of bowel ischemia [40] or to assess small bowel lesions in Crohn’s disease [48] and gastrointestinal stromal tumours as reported by Martin et al [49].

The increase of the f_av_ values with DLSR V2 offers the possibility to use the strong level to increase the detectability of lesions with low iodine concentration and to maximise the potential iodine reduction. For example, the f_av_ value at 60 keV with DLSR V2 was close to that obtained with DLSR V1 at the standard level. It would therefore be possible to consider replacing the default standard level setting in DLSR V1 by the strong level in version V2, without major modification in image texture. Finally, these results on phantoms need to be confirmed by a study on patients.

This study has some limitations. First, we explored only one dose level and one phantom size. However, the dose level was chosen to correspond to the standard level used in clinical applications for most patients. Second, we used a single reconstruction kernel and only one slice thickness, as explained earlier. Third, we used a phantom that, by its very nature, does not take into account the patient movements during CT scanning. We assessed only one DECT platform because the DLSR algorithm was not available on other Canon DECT platforms. Also, these results were not compared to other reconstruction methods because these methods are not available on this platform (Aquilion ONE PRISM). Finally, the size of the samples compared for some results is small, limiting the power of the statistical analysis which may explain why some differences resulted to be not significant.

In conclusion, the new version of DLSR reduces the noise magnitude, improves noise texture, increases spatial resolution, and detectability of low iodine concentration in VMIs. These findings suggest a great potential of the new version of DLSR for reducing the amount of injected iodine to the patients at the standard radiation dose.

## Supplementary Information

**Additional file 1.**

## Data Availability

The datasets analysed during the current study are available from the corresponding author on reasonable request.

## Abbreviations

ACR: American College of Radiology
CT: Computed tomography
d': Detectability index
DECT: Dual-energy CT
DL: Deep learning
DLSR: DL spectral reconstruction
f_av_: Average NPS frequency
NPS: Noise power spectrum
ROI: Region of interest
SECT: Single-energy CT
TTF: Task-based transfer function
TTF_50_: Task-based transfer function at 50 %
VMIs: Virtual monoenergetic images
