# Supplementary material for: Comparison of image quality of two versions of deep-learning image reconstruction algorithm on a rapid kV-switching CT: a phantom study
Source: Eur Radiol Exp. 2023 Jan 9;7:1. doi: 10.1186/s41747-022-00314-9 (PMC9826773; doi:10.1186/s41747-022-00314-9)
Supplement: Supplementary file 1 — Additional file 1. [file 41747_2022_314_MOESM1_ESM.docx]

**ELECTRONIC SUPPLEMENTARY MATERIAL**

**Comparison of image quality of two versions of deep-learning image reconstruction algorithm on a rapid kV-switching CT: a phantom study**

**Materials and methods**

***Deep learning spectral reconstruction process***

A rapid kV switching CT is based on the acquisition of two X-ray spectra at low and high kV by switching between low and high kV during acquisition. Then, spectral image reconstruction takes advantage of the fact that a large part of the information contained in a high kV view and a low kV view at a particular location is common to both views. The difference between high and low energy views is the degree to which the X-ray beam is attenuated by the patient. This process is based on transforming the views of one energy into another to create deep learning views (DLV).These DLV are produced by the trained neural network using the measured data from views of the opposite energy and adjacent views of the same energy. The DLVs compliment the measured views at each energy to generate a complete sinogram for each kV. DLSR was trained on complete measured sinograms acquired at each kV for a wide variety of patient and phantom attenuation levels. The sinogram data used for training were processed with an array of sophisticated models. After the extensive training process, Spectral Reconstruction was tested with independent validation datasets. Then engineers, medical physicists, and radiologists reviewed image results extensively.

The figure (1 SM) shows the steps of deep learning spectral reconstruction (DLSR) process.


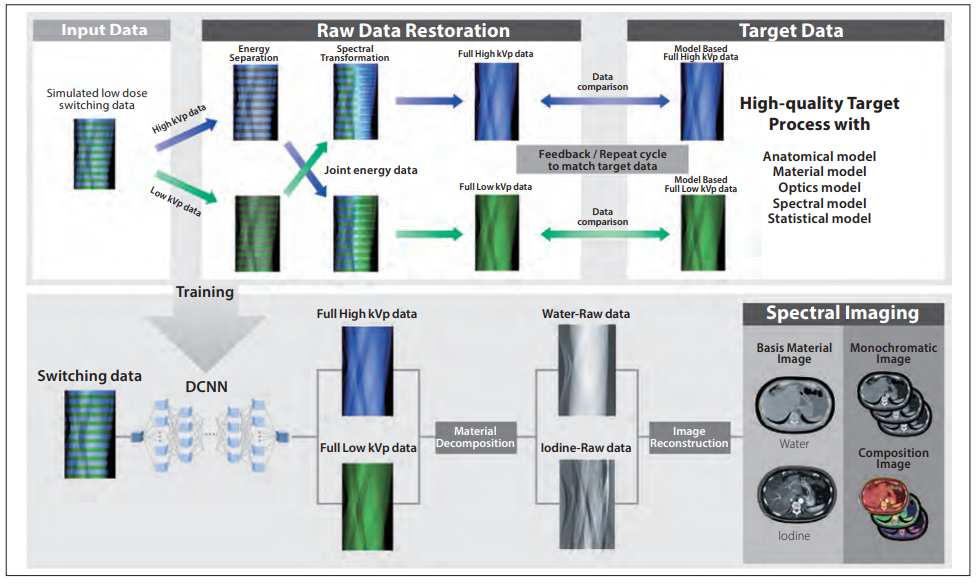


Figure (1 SM): Deep learning spectral reconstruction process [1 SM].

***Contrast to noise ratio (CNR)***

The contrast-to-noise ratio values were calculated using for three iodine inserts at 2, 1 and 0.5 mg/mL for both DLSR versions according to four energy levels (40/50/60/70 keV) and DLSR levels (Mild, Standard and Strong). CNR was calculated using a following formula (SM 1):

$CNR=\frac{{HU}_{iodine}-{HU}_{solid water}}{\sigma_{solid water}}$ (1 SM)

Where, ${HU}_{iodine}$corresponds to the mean HU value of each iodine insert (0.5, 1 and 2 mg/mL) and ${HU}_{solid water}$ to the solid water value, $\sigma_{solid water}$ is the noise value measured in the solid water.

**Results**

We observed that for both versions, CNR increased significantly with DLSR levels except between Mild and Standard levels at 60 keV with DLSR V1 where similar values were found (*p* = 0.761).

For DLSR V1, the CNR values increased significantly between 40 and 70 keV for all iodine inserts. In the case of DLSR V2, the CNR values peaked at 60 keV and decreased significantly between 60 and 70 keV except for 0.5 mg/mL insert where the mean difference was of 12.6%±1.6% (*p* = 0.230).

The CNR values were significantly higher with DLSR V2 for iodine insert of 2 mg/mL. For iodine insert of 1 mg/mL, this difference between DLSR V2 and DLSR V1 was not significant with Strong level at 40 (2.9%± 1.1%, *p* = 0.518) and 60 keV (-1.6%±0.7%, *p* =0.250). For the insert of 0.5 mg/mL, the difference was not significant at 50 keV (15.1%±4.3%, *p* =0.342), 60 keV (6.25%±3.2%, *p* =0.280) for Strong level and at 70 keV for all DLSR levels.

Figures (SM2), (SM3) and (SM4) depicted the variation of CNR as function of energy level, DLSR level and version for iodine inserts of 2 mg/mL, 1 mg/mL and 0.5 mg/mL respectively.

**
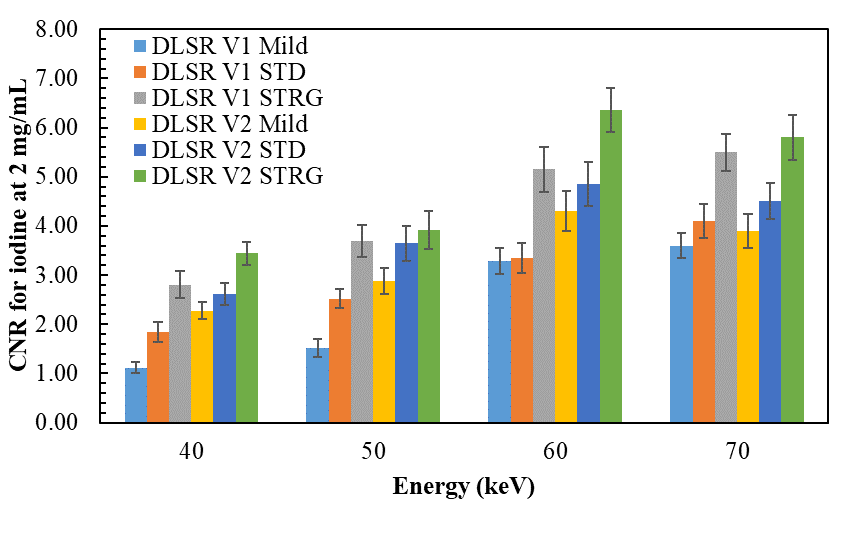
**

**Figure (2 SM) :** variation of CNR values as function of energy level, DLSR level and version for iodine insert of 2 mg/mL

**
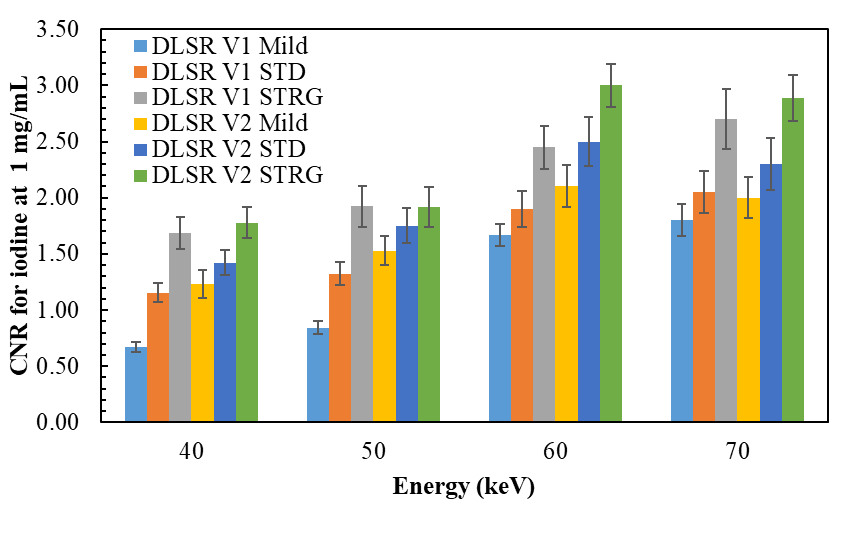
**

**Figure (3 SM):** variation of CNR values as function of energy level, DLSR level and version for iodine insert of 1 mg/mL

**
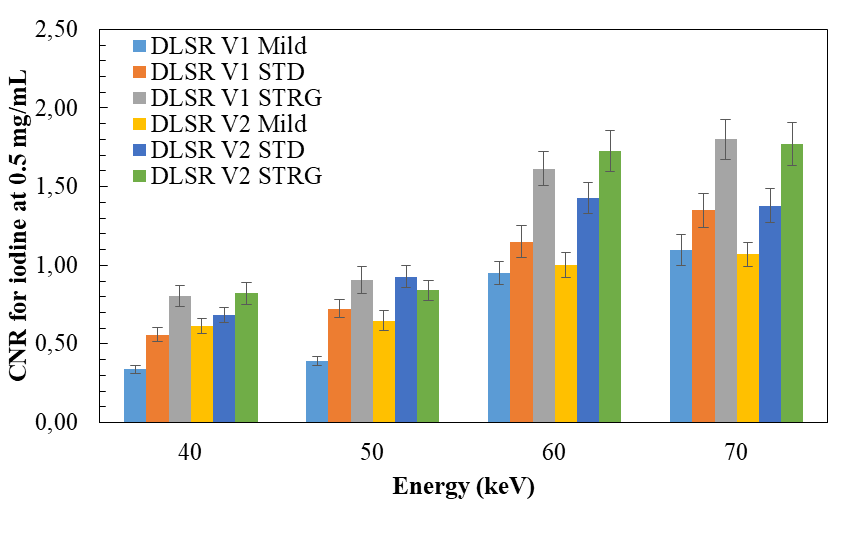
**

**Figure (4 SM):** variation of CNR values as function of energy level, DLSR level and version for iodine insert of 0.5 mg/mL

**Reference**:

[1 SM] Boedeker K, Hayes M, Zhou J, Zhang R, Yu Z (2019) Deep Learning Spectral CT – Faster, easier and more intelligent. Whitepaper - Canon Medical Systems. Accessed 2019-12
